# Associations between BMI trajectories and young adult bone: a 10-yr follow-up from the Norwegian Fit Futures cohort

**DOI:** 10.1093/jbmrpl/ziag143

**Published:** 2026-08-25

**Authors:** Marianne Lund, Anja D Norbye, Anne-Sofie Furberg, Jonas Johansson, Tom Wilsgaard, Anne Winther, Tore Christoffersen, Elin Evensen

**Affiliations:** Department of Health and Care Sciences, Faculty of Health Sciences, UiT The Arctic University of Norway, 9019 Tromsø, Norway; Department of Health and Care Sciences, Faculty of Health Sciences, UiT The Arctic University of Norway, 9019 Tromsø, Norway; Faculty of Health and Social Sciences, Molde University College, 6410 Molde, Norway; Department of Microbiology and Infection Control, University Hospital of North-Norway, 9038 Tromsø, Norway; Department of Community Medicine, Faculty of Health Sciences, UiT The Arctic University of Norway, 9019 Tromsø, Norway; Department of Community Medicine, Faculty of Health Sciences, UiT The Arctic University of Norway, 9019 Tromsø, Norway; Division of Medicine, University Hospital of North Norway, 9038 Tromsø, Norway; School of Sports Sciences, Faculty of Health Sciences, UiT The Arctic University of Norway, 9019 Tromsø, Norway; Research and Development Department, Finnmark Hospital Trust, 9510 Alta, Norway; Department of Health and Care Sciences, Faculty of Health Sciences, UiT The Arctic University of Norway, 9019 Tromsø, Norway

**Keywords:** BMD, BMC, adolescence, young adulthood, BMI, BMI-trajectories, population-based study, DXA, skeletal development

## Abstract

BMI is a well-established determinant of bone health; however, most evidence is derived from cross-sectional or short-term studies, providing limited insight into how long-term BMI development influences skeletal maturation. We aimed to investigate the associations between long-term BMI trajectories and skeletal development from adolescence into young adulthood. Data were derived from 1114 participants in the population-based Fit Futures study with repeated measurements (2010-2011 [FF1], 2012-2013 [FF2], and 2021-2022 [FF3]). Group-based trajectory modeling identified 3 BMI trajectories: low-stable, moderate-increasing, and high-stable. Sex-stratified linear mixed-effects models estimated adjusted mean DXA-derived areal BMD (aBMD) and BMC at the total body (TB), TH, and FN. Participants in the moderate-increasing and high-stable trajectories consistently exhibited higher aBMD and BMC than those in the low-stable trajectory across all skeletal sites throughout follow-up. For TB aBMD, compared with the low-stable trajectory, the moderate-increasing trajectory was associated with β = .06 g/cm^2^ (95% CI: 0.05-0.08) in females and β = .08 g/cm^2^ (95% CI: 0.06-0.10) in males, whereas the corresponding estimates for the high-stable trajectory were β = .10 g/cm^2^ (95% CI: 0.07-0.17) and β = .10 g/cm^2^ (95% CI: 0.06-0.15), respectively (all *p* < .001). Despite continued increases in BMI, the moderate-increasing trajectory was not associated with proportionally greater skeletal accrual than the high-stable trajectory, and statistically significant trajectory-by-time interactions were limited to TB outcomes. BMI trajectories established during adolescence were associated with persistent differences in attained skeletal levels rather than differential skeletal accrual into young adulthood. These findings underscore the importance of adolescence as a critical period for the attainment of peak bone mass.

## Graphical Abstract

**Graphical Abstract:**
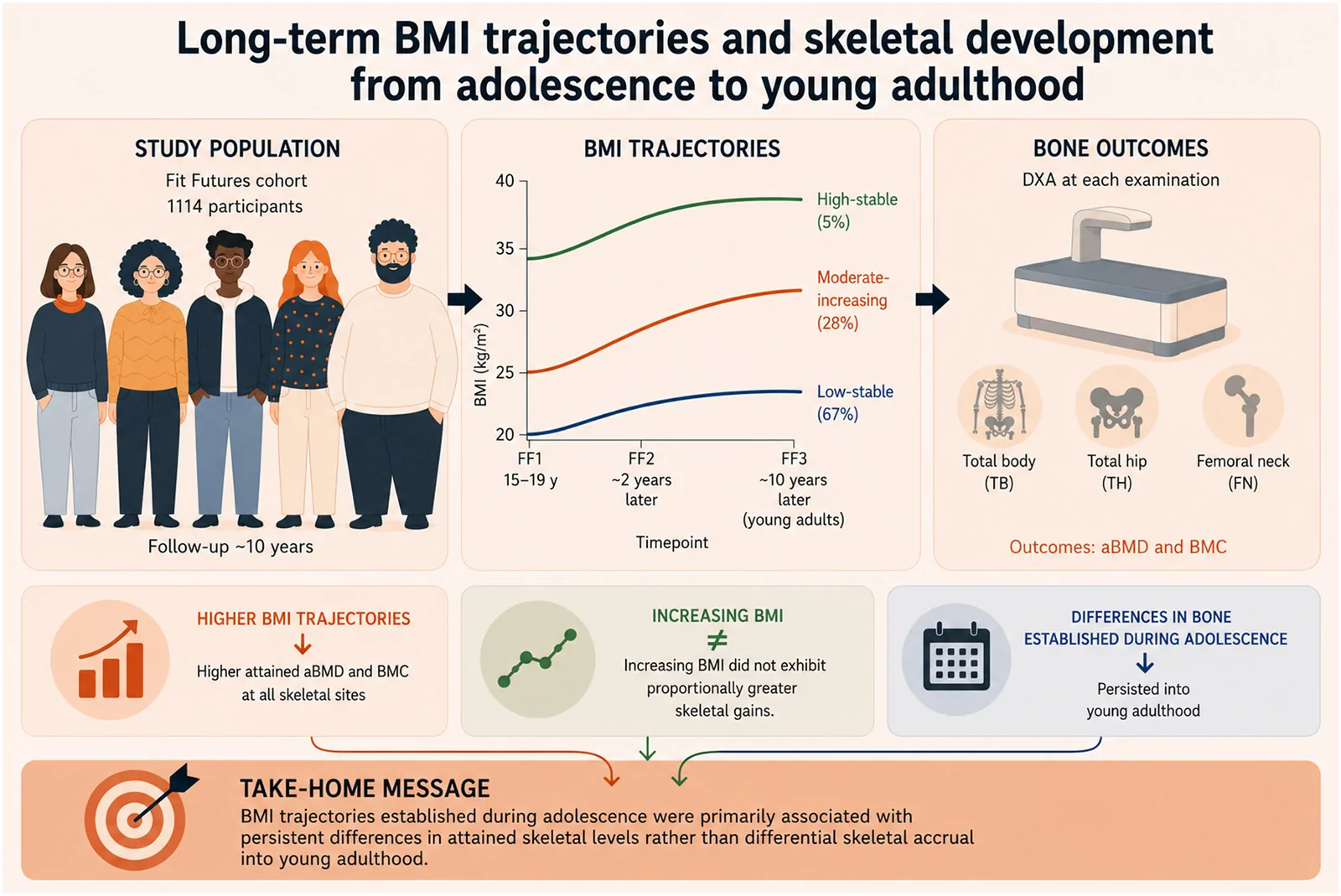

## Introduction

Peak bone mass (PBM) refers to the maximal amount of bone tissue present at the end of skeletal maturation and is typically attained in late adolescence or early adulthood.^1^^,^^2^ Timing and level of PBM vary by sex, age, pubertal maturation, and skeletal site.^2^^,^^3^ While genetic factors are the primary determinants of PBM,^4^ modifiable lifestyle factors account for 20%-40% of the variation,^1^^,^^3^ and even small improvements in bone acquisition during growth may influence PBM attainment,^2^ which is a major determinant of osteoporosis risk later in life.^1^^,^^5^ Adolescence and early adulthood constitute a critical developmental period marked by rapid changes in growth, body composition, and hormonal regulation,^6^ making these years particularly responsive to factors that influence bone acquisition.^1^

Lifestyle-related factors such as body composition and adiposity influence bone development through both mechanical and metabolic pathways.^7^ As bone adapts to habitual mechanical stimuli,^8^ higher body mass may promote mineral accumulation through greater skeletal loading. However, this relationship is complex, as greater adiposity may simultaneously exert adverse metabolic effects during growth, including inflammation and endocrine alterations associated with impaired bone quality and increased fracture risk in children and adolescents.^9^

BMI, a widely used measure of body size in epidemiological research, is often employed to capture these adiposity-related influences.^10^ While observational studies generally report positive associations between BMI and areal BMD (aBMD),^11^^,^^12^ higher BMI has also been linked to increased fracture risk,^13^ suggesting that factors, such as lean mass, fat distribution, and bone quality, may influence this relationship.^9^ Conversely, low BMI is associated with reduced mechanical loading and lower nutritional intake, both of which are linked to lower bone mass.^14^^,^^15^

The dual and sometimes opposing influences highlight the need to clarify how BMI relates to bone development during adolescent years, in which PBM is formed. However, evidence remains limited,^11^ as most existing studies are cross-sectional, and longitudinal studies are typically short-term,^15^ providing little insight into bone developmental patterns during a period characterized by changes in growth and body composition. Furthermore, prior research has often been constrained by single-sex samples, narrow age ranges, or the absence of repeated DXA assessments across adolescence.^11^^,^^16–18^ As a result, longitudinal evidence spanning early adolescence to young adulthood and capturing sustained BMI patterns across this developmental window remains scarce.^1^^,^^3^^,^^19^^,^^20^ In addition, trajectory-based approaches, which may offer a more comprehensive characterization of long-term BMI exposure in relation to bone mineral accrual,^21^ have rarely been applied.

To our knowledge, no population-based longitudinal cohort has included repeated DXA measurements in both sexes from early adolescence into young adulthood. To address this gap, we used data from the Fit Futures study, a population-based cohort with repeated DXA assessments across this period.

The aim of this 10-yr longitudinal study was to investigate whether associations between BMI and bone outcomes established during adolescence persist into young adulthood. Building on previous findings from the Fit Futures study,^15^^,^^22^ and drawing upon the mechanostat theory linking mechanical loading to bone mineral accrual, we hypothesized that higher BMI trajectories would be associated with higher aBMD, with the possibility of non-linear associations and plateauing at higher BMI levels.

## Materials and methods

### Study design and population

The Fit Futures study is a population-based study from Northern Norway, following participants from adolescence into young adulthood through 3 repeated health surveys. The median follow-up time from baseline (FF1) to FF3 was 10.5 yr (range: 9.9-11.4).

In 2010-2011, all first-year students from all upper secondary schools in Tromsø and the neighboring municipality were invited to participate in the first Fit Futures study (FF1). In total, 1038 girls and boys attended the survey (attendance: 93%).

The second survey (FF2) was conducted in 2012-2013. All participants from FF1 were invited to FF2. In addition, all third-year students attending the same upper secondary schools during FF2 were invited, including individuals who had declined participation in FF1 and newly admitted students. In total, 868 individuals participated in FF2, of whom 133 were new to the study (attendance: 74%).

A third data collection phase, FF3, was conducted between 2021 and 2022. All individuals from the original cohort, who had previously participated in FF1 and/or FF2 were invited. Seven hundred and sixteen participants were included in FF3 (attendance: 62%).

The study has an open cohort design, allowing for new participants at FF2 and intermittent participation across waves. Participants were not required to attend all 3 surveys to be included in the present study. The analytical sample comprised all unique participants with valid data from at least one survey wave, resulting in an unbalanced longitudinal dataset in which individuals could contribute data from 1, 2, or all 3 waves. In total, 1114 unique participants were included in the analytic sample. Of these, 219 (19.7%) contributed data from 1 wave only (FF1 only: 150; FF2 only: 69), 388 (34.8%) from 2 waves (FF1-FF2: 210; FF2-FF3: 64; FF1-FF3: 114), and 507 (45.5%) from all 3 waves.

**Figure 1:**
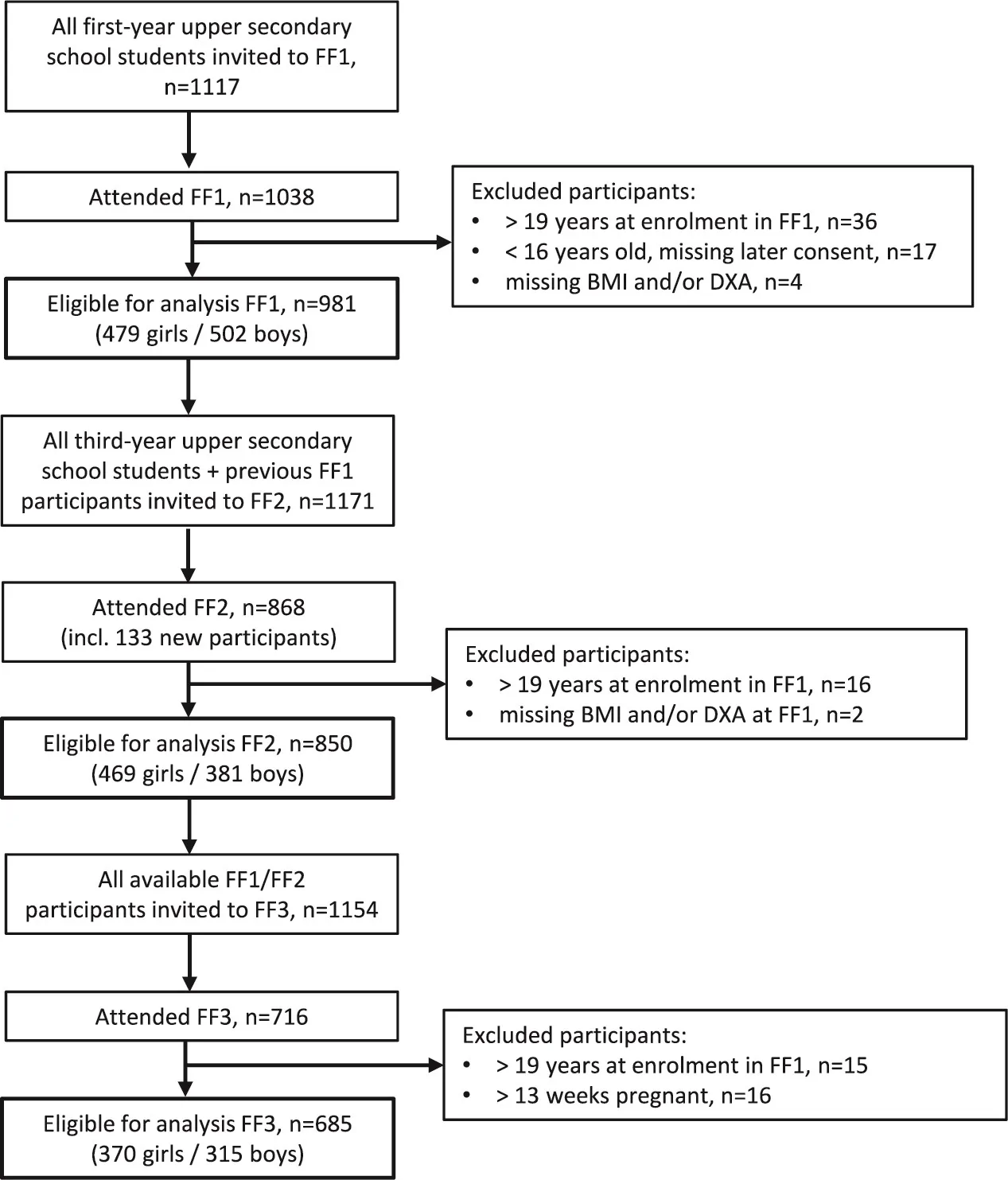
Flowchart illustrating participant inclusion and follow-up from FF1 to FF3 in the Fit Futures study. Wave-specific exclusions are shown for each examination.

A flowchart of recruitment, follow-up, and exclusions is presented in Figure 1.

### Ethics

The present study was approved by the Regional Committee for Medical and Health Research Ethics, North Norway (ID 758656/REK Nord). The Fit Futures study (ID 2009/1282/REK Nord) has been conducted in accordance with the Norwegian Regulations on Population-Based Health Research and applicable data protection legislation.^23^ The study adheres to national guidelines for ethical conduct and the protection of privacy in health research.^24^^,^^25^ Participation is voluntary, and written informed consent was obtained from all participants at each of the 3 data collection waves. Participants younger than 16 yr at baseline were enrolled with additional parental consent. Upon reaching the age of consent, these participants were required to provide their own consent for the continued use of data from FF1. Seventeen of these participants did not participate in a subsequent follow-up and later attempts to obtain their consent were unsuccessful. Consequently, their data were not available for the present study.

### Measurements

Trained research staff at the Clinical Research Unit at the University Hospital of North Norway performed all clinical examinations at all 3 waves according to standardized protocols. Information on diseases, medication use, contraception, menstruation, and pregnancy was collected through clinical interviews. Lifestyle-related factors were obtained through self-administered electronic questionnaires developed for the Fit Futures study. Standardized instruments used for specific measures are described below.

### Outcome measures

DXA measurements were performed to assess aBMD (g/cm^2^) and BMC (g) at the FN, TH, and in the total body (TB). Trained technicians following a standardized protocol performed the measurements using a GE Lunar Prodigy scanner (GE Healthcare). Quality controls were performed on a daily basis according to the manufacturer’s procedure. The in vivo coefficients of variation (CVs) for the DXA machine used in this study have previously been estimated to be 1.17% for TH and 1.72% for FN BMD measurements.^26^ No specific precision assessment was performed for the Fit Futures study. Baseline age- and sex-specific aBMD Z-scores for the TB, TH, and FN were obtained directly from the GE Lunar enCORE software using the manufacturer-provided reference database.^27^

The left hip served as the reference site for all hip measurements across all examinations to ensure standardization throughout the study waves; however, in 13 participants, data from the left side were erroneous or missing. In these cases, right hip measurements were consistently used across measurement points.

All DXA scans from the same examination wave were analyzed by a single investigator.

### Exposure variables

Body weight and height were measured using an automatic electronic scale with integrated stadiometer (Jenix DS 102, Dong Sahn Jenix). Body weight was measured to the nearest 0.1 kg, with participants wearing only light clothing and no shoes. Standing height was measured to the nearest 0.1 cm. BMI was calculated as weight (kg) divided by height squared (m^2^), following established anthropometric methodology. Classification of BMI was based on the WHO reference standards for adults.^28^ For participants aged 15-18 yr, BMI values were classified according to the International Obesity Task Force cut-offs.^29^

### Covariates and potential confounders

Covariates considered potential confounders were selected a priori based on previous literature documenting their associations with BMI and bone outcomes.^1^^,^^30^ Variables included in the models to account for biological and modifiable lifestyle factors known to influence bone accrual and metabolism included age, pubertal maturation, physical activity, tobacco use, alcohol consumption, and, among females, hormonal contraceptive use. Although not a lifestyle factor per se, serum vitamin D reflects modifiable exposure related to sunlight, diet, and vitamin D supplements, and was therefore included as a covariate. Height was considered a potential covariate but was not included to avoid inappropriate adjustment due to mathematical coupling, as height is a component of BMI. Ethnicity was tested as a potential covariate but was excluded from the final model, as inclusion did not materially influence the model estimates. This likely reflects the ethnic homogeneity in the study population (97% White).

Pubertal maturation was assessed using sex-specific measures. In females, self-reported age at menarche was categorized as early (<12.5 yr), intermediate (12.5-13.9 yr), or late (≥14 yr). In males, pubertal stage was evaluated using the Pubertal Development Scale (PDS), comprising self-ratings of 4 secondary sexual characteristics on a 4-point Likert scale.^31^ The mean PDS score was used as a continuous indicator of maturation and further categorized as “not yet begun,” “barely started,” “underway,” or “completed.”

Serum 25OHD concentrations from non-fasting blood samples were measured by liquid chromatography–tandem mass spectrometry (LC-MS/MS). Samples from FF1 and FF2 were analyzed at the Hormone Laboratory, Haukeland University Hospital, whereas FF3 samples were analyzed using the same method at the Department of Laboratory Medicine, University Hospital of North Norway. Further details of the analytical procedure have been described previously.^32^ Concentrations were classified according to international consensus guidelines as severe deficiency (<30 nmol/L), insufficiency (30-49.9 nmol/L), sufficiency (50-74.9 nmol/L), or optimal (≥75 nmol/L).^33^

Physical activity was assessed using a modified version of the Saltin-Grimby Physical Activity Level Scale,^34^ with 4 categories reflecting average leisure-time activity during the past year: sedentary, moderate (light activity ≥4 h/wk), sport (hard training several times per week), and competition (strenuous training or competitive sports multiple times per week).

Smoking and snuff use were initially assessed as never, occasional, or daily; alcohol consumption was reported across 5 frequency categories from never to ≥4 times/wk. For analysis, tobacco use (smoking and snuff combined) and alcohol use were dichotomized into any vs none.

Hormonal contraceptive use in females was categorized as none, combined estrogen-progestin, or progestin-only, and further dichotomized into any use vs none to ensure adequate subgroup sizes and model stability.

### Statistical analysis

All analyses were stratified by sex to account for known differences in pubertal timing, hormonal influences, growth patterns, and PBM trajectories between males and females.^35^

To capture trajectories of BMI, we employed group-based trajectory modeling using the *traj* plugin in Stata.^36^ This approach identifies latent subgroups of individuals following similar developmental trajectories over time. BMI was analyzed as a continuous variable using the censored normal (*cnorm*) distribution, appropriate for variables with natural lower bounds.^36^ Models specifying 2-5 latent trajectories were estimated. With 3 measurement occasions, the maximum identifiable polynomial order is quadratic (degree = number of timepoints minus one), and quadratic terms were therefore specified accordingly.^37^ Model selection was guided by the Bayesian information criterion, entropy, group size, and theoretical interpretability, with the optimal model reflecting the best balance between statistical fit and biological plausibility.^37^ Posterior probabilities were inspected to confirm adequate classification accuracy, and the participants were assigned to the trajectory group with the highest probability.^36^

The final trajectory model comprised 3 BMI trajectory groups (low-stable, moderate-increasing, and high-stable). A 3-group model with quadratic polynomial terms (2 2 2) provided the best overall fit. Entropy for the selected model was 0.81, indicating good classification accuracy, and all trajectory groups exceeded the recommended minimum size threshold of 5%.^21^ Classification quality was further supported by high average posterior probabilities for all groups (0.93, 0.86, and 0.93, respectively). Overall, 88.4% of participants had a maximum posterior probability ≥0.70, and sensitivity analyses restricted to these participants yielded similar results.

Baseline was defined as FF1, and baseline characteristics are presented for participants attending this examination, stratified by BMI trajectory group (low-stable, moderate-increasing, and high-stable). Participants entering the study at FF2 were not included in these baseline descriptives. Continuous variables are presented as means and SD, and categorical variables as frequencies and percentages. Group differences were assessed using one-way ANOVA for continuous variables and chi-square tests for categorical variables. Assumptions of normality and homogeneity of variances were evaluated using visual inspection of histograms and Q–Q plots and Levene’s test, respectively. For chi-square analyses, expected cell counts were examined to ensure validity. All assumptions were met.

Associations between BMI trajectories and bone outcomes (aBMD and BMC at the FN, TH, and TB) were examined in 2 stages using linear mixed-effects models estimated by maximum likelihood with random intercepts for participant ID to account for within-subject correlation.^38^ First, repeated measurements from FF1 to FF3 were analyzed using sequentially adjusted models:

- **Model 0:** crude.
- **Model 1:** adjusted for biological factors (baseline age (FF1), pubertal maturation).
- **Model 2:** additionally adjusted for modifiable lifestyle factors (physical activity, tobacco use, alcohol consumption, serum vitamin D level, and hormonal contraceptive use in females).

Second, to examine changes in associations over time, longitudinal mixed models including BMI trajectory-by-time (FF1-FF3) interaction terms were fitted separately for TB, TH, and FN and fully adjusted for relevant biological and lifestyle covariates (Model 2). Statistical significance of the trajectory × time interaction was assessed using joint Wald tests.

Linear mixed-effects models allowed inclusion of all available observations under the assumption that outcome data were missing at random (MAR), without requiring complete data across all timepoints. Model assumptions were assessed by inspecting residual plots for linearity, homoscedasticity, and normality, and the assumptions were deemed adequately met.

Regression coefficients (β) with 95% CIs are reported for associations between BMI trajectories and bone outcomes. Model-based adjusted means derived from marginal predictions, together with their corresponding 95% CIs, were used for descriptive and illustrative presentation of longitudinal patterns. A 2-sided significance level of α = .05 was applied. All analyses were conducted in Stata version 18 (StataCorp LLC).

Missing covariate data reduced the number of observations available for the sequentially adjusted models. Small differences in the number of observations between outcome measures reflected occasional missing outcome data. Among boys, pubertal status (PDS) was missing for 21% of observations because the questionnaire was introduced after data collection had commenced. In addition, baseline age (FF1) was unavailable for some participants and was therefore estimated from age at FF2 to retain these individuals in the analyses.

To assess the robustness of the findings, sensitivity analyses were performed. First, boys with available PDS at FF1 were compared with those with missing PDS regarding baseline age, BMI, and bone outcomes using independent-samples *t*-tests. Second, participants entering the study at FF2 were compared with those present at baseline (FF1) regarding BMI and bone outcomes at FF2 using independent-samples *t*-tests.

## Results

### Descriptive data

At baseline, the study included 981 adolescents (48.8% girls and 51.2% boys) aged 15-19 yr (mean [SD]: 16.2 [0.6]).

Based on repeated BMI measurements across FF1-FF3, 3 BMI trajectory groups were identified (low-stable, moderate-increasing, and high-stable). Baseline (FF1) characteristics of participants according to these trajectory groups are presented below. The low-stable trajectory comprised the majority of participants (girls: 70%, *n* = 331; boys: 67%, *n* = 334) and included individuals classified as underweight or normal weight at baseline; 92.5% of girls and 90% of boys in this group were of normal weight at FF1. The moderate-increasing trajectory included 24% of girls (*n* = 117) and 28% of boys (*n* = 143) and was characterized by a sustained increase in BMI over time. At baseline, 51.3% of girls and 60% of boys in this group were classified as overweight, while 40.2% of girls and 30.8% of boys were of normal weight. The high-stable trajectory was the smallest group (girls: 6%, *n* = 31; boys: 5%, *n* = 25) and was characterized by high BMI already at baseline, corresponding predominantly to obesity, with persistently high BMI throughout follow-up.

Overall, BMI tracking within clinical categories was strong in the low-stable and high-stable trajectories, whereas the moderate-increasing trajectory captured individuals transitioning toward higher BMI categories during follow-up. Across trajectory groups, body weight differed as expected by design, whereas height and age were similar between groups in both sexes.

Pubertal maturation differed across BMI trajectory groups in both sexes (girls: *p* = .001; boys: *p* = .022). Among girls, early pubertal timing was most frequent in the moderate-increasing trajectory, whereas among boys, completed pubertal development was most frequent in this group. Vitamin D status differed across trajectory groups in both sexes, with severe deficiency most frequent in the high-stable trajectory (girls: *p* = .020; boys: *p* = .018). Lifestyle characteristics differed only among girls, in whom physical activity and alcohol consumption varied across BMI trajectories, whereas no statistically significant differences in physical activity, tobacco use, or alcohol consumption were observed among boys.

Baseline aBMD *Z*-scores increased progressively from the low-stable to the moderate-increasing and high-stable trajectories at all skeletal sites in both females and males (all *p* < .001), indicating that participants in the higher BMI trajectories had higher age- and sex-standardized BMD already at baseline.

**Figure 2:**
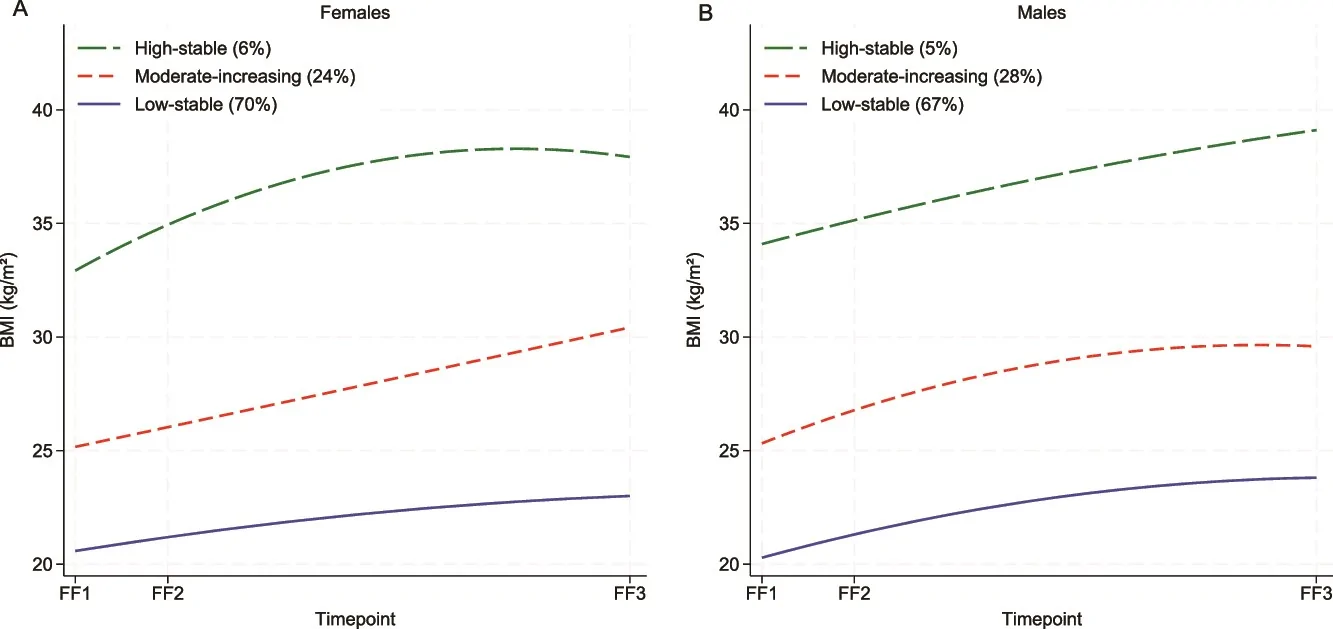
BMI trajectory patterns from FF1 to FF3 in the Fit Futures study, stratified by sex: (A) females and (B) males.

BMI trajectory patterns are presented in Figure 2, and baseline characteristics stratified by sex and BMI trajectory group are shown in Tables 1 and 2. Descriptive TB lean and fat mass according to BMI trajectory group at each examination are presented in Table S1.

**Table 1:** Baseline characteristics of girls according to BMI trajectory group, the Fit Futures study.

Variable	Total (*n* = 479)	Low-stable (*n* = 331)	Moderate-increasing (*n* = 117)	High-stable (*n* = 31)	*p*
**Age (yr)**	16.2 ± 0.6	16.2 ± 0.6	16.2 ± 0.7	16.4 ± 0.7	.27
**Ethnicity, White**	96.5	97.3	94	96.8	.26
**Height (cm)**	164.7 ± 6.6	164.9 ± 6.3	164.4 ± 6.8	163.4 ± 8.9	.42
**Weight (kg)**	61 ± 11.8	56 ± 6.2	68.1 ± 9.3	88.1 ± 15	**<.001**
**BMI (kg/m** ^**2**^ **)**	22.5 ± 4.1	20.6 ± 1.8	25.2 ± 2.7	32.9 ± 4.7	**<.001**
**Pubertal timing, *n* = 467**					**.001**
**Early (<12.5 yr)**	31.9	28.5	41.8	32.3	
**Intermediate (12.5-13.9 yr)**	45.6	45.4	49.1	35.5	
**Late (≥14.0 yr)**	22.5	26.1	9.1	32.3	
**Vitamin D (nmol/L), *n* = 420**					**.02**
**Severe deficiency (<30 nmol/L)**	14.1	12.3	15.5	28	
**Insufficiency (30-49.9 nmol/L**	34.5	34.3	31.1	52	
**Sufficiency (50-74.9 nmol/L)**	32.1	31.5	36.9	20	
**Optimal (≥75 nmol/L)**	19.3	21.9	16.5	-	
**Physical activity, *n* = 474**					**.047**
**Sedentary**	13.9	14.6	10.6	19.4	
**Moderate**	40.5	36.1	50.4	51.6	
**Sports**	29.3	30.9	25.7	25.8	
**Competition**	16.2	18.5	13.3	3.2	
**Tobacco use (yes), *n* = 472**	39.6	38.6	43.8	35.5	.56
**Alcohol consumption (yes), *n* = 474**	76.4	78.5	75.2	58.1	**.036**
**Hormonal contraceptives (yes), *n* = 182**	94.5	95.7	94.1	85.7	.30
**aBMD, g/cm** ^**2**^					
**Total body**	479	1.12 ± 0.07	1.18 ± 0.06	1.21 ± 0.06	**<.001**
**Total hip**	478	1.03 ± 0.12	1.11 ± 0.11	1.15 ± 0.11	**<.001**
**Femoral hip**	479	1.04 ± 0.12	1.12 ± 0.11	1.15 ± 0.10	**<.001**
**BMD, Z-score**					
**Total body**	468	−0.07 ± 0.93	0.73 ± 0.85	1.07 ± 0.82	**<.001**
**Total hip**	468	0.18 ± 0.92	0.73 ± 0.79	1.03 ± 0.79	**<.001**
**Femoral hip**	469	0.31 ± 0.88	0.84 ± 0.80	1.04 ± 0.71	**<.001**

Baseline = FF1; new participants entering at FF2 are not included.

*p*-values obtained by one-way ANOVA for continuous variables and χ^2^ tests for categorical variables. *p*-values in bold indicate statistical significance at *p* < 0.05. Data are presented as mean ± SD or % unless otherwise stated.

Pubertal timing based on self-reported age of menarche. Physical activity based on self-reported scores on the Saltin-Grimsby Physical Activity Level Scale.

BMD Z-scores were generated by the Lunar DXA software using age- and sex-specific reference data and were only available for participants aged 15-18 yr.

**Table 2:** Baseline characteristics of boys according to BMI trajectory group, the Fit Futures study.

Variable	Total (*n* = 502)	Low-stable (*n* = 334)	Moderate increasing (*n* = 143)	High-stable (*n* = 25)	*p*
**Age (yr)**	16.2 ± 0.6	16.2 ± 0.6	16.2 ± 0.7	16.4 ± 0.8	.27
**Ethnicity, White**	96.8	97.3	96.50	92	.37
**Height (cm)**	177 ± 6.6	177.1 ± 6.6	176.5 ± 6.7	179.6 ± 5.5	.09
**Weight (kg)**	70.4 ± 14.4	63.7 ± 7.9	79 ± 9.8	110 ± 13.2	**<.001**
**BMI (kg/m** ^**2**^ **)**	22.4 ± 4.1	20.3 ± 2	25.3 ± 2.4	34.1 ± 4.1	**<.001**
**Pubertal stage, *n* = 397**					**.022**
**Completed (PDS 4.0)**	9.8	6.6	17.3	12.5	
**Underway (PDS 3.0-3.9)**	73.1	76.8	63.6	75	
**Barely started (PDS 2.0-2.9)**	17.1	16.6	19.1	12.5	
**Vitamin D (nmol/L), *n* = 468**					**.018**
**Severe deficiency (<30 nmol/L)**	39.5	35.6	43.9	71.4	
**Insufficiency (30-49.9 nmol/L**	29.9	31.1	28.0	23.8	
**Sufficiency (50-74.9 nmol/L)**	24.2	25.4	24.2	4.8	
**Optimal (≥75 nmol/L)**	6.4	7.9	3.8	-	
**Physical activity, *n* = 495**					.17
**Sedentary**	29.3	28	29.8	44	
**Moderate**	25.3	23.7	29.1	24	
**Sports**	22.6	22.8	21.3	28	
**Competition**	22.8	25.5	19.9	4	
**Tobacco use (yes), *n* = 494**	44.5	42.6	47.1	56	.33
**Alcohol consumption (yes), *n* = 493**	76.4	67.2	69.8	76	.60
**aBMD, g/cm** ^**2**^					
**Total body**	502	1.16 ± 0.09	1.22 ± 0.09	1.26 ± 0.07	**<.001**
**Total hip**	502	1.09 ± 0.14	1.15 ± 0.16	1.19 ± 0.12	**<.001**
**Femoral neck**	502	1.08 ± 0.14	1.15 ± 0.16	1.18 ± 0.15	**<.001**
**BMD, *Z*-score**					
**Total body**	496	0.02 ± 0.92	0.72 ± 0.90	1.03 ± 0.81	**<.001**
**Total hip**	496	−0.06 ± 0.99	0.47 ± 1.11	0.61 ± 0.94	**<.001**
**Femoral neck**	496	−0.10 ± 1.05	0.49 ± 1.16	0.63 ± 1.18	**<.001**

Baseline = FF1; new participants entering at FF2 are not included.

*p*-values obtained by one-way ANOVA for continuous variables and χ^2^tests for categorical variables. *p*-values in bold indicate statistical significance at *p* < 0.05. Data are presented as mean ± SD or % unless otherwise stated.

Pubertal stage based on self-rated scores on the Pubertal Development Scale (PDS). Physical activity based on self-reported scores on the Saltin-Grimsby Physical Activity Level Scale.

BMD Z-scores were generated by the Lunar DXA software using age- and sex-specific reference data and were only available for participants aged 15-18 yr.

### Sensitivity analyses

To evaluate potential bias related to missing PDS in boys, baseline characteristics were compared between boys with and without available PDS in FF1. No statistically significant differences were observed in BMI, FN or TH aBMD (all *p* > .005). However, boys with available PDS data were slightly older (mean diff. 0.15 yr, 95% CI [0.02, 0.29], *p* = .023) and exhibited marginally higher TB aBMD (mean diff. 0.023 [0.002, 0.043], *p* = .031) compared to those with missing PDS data.

The sensitivity analysis comparing participants who entered the study at FF2 with those who were present at baseline revealed no statistically significant difference in age, BMI, or bone outcomes at FF2 (all *p* > .005).

### Associations between BMI trajectory and bone outcomes

Both the moderate-increasing and the high-stable BMI trajectories were associated with higher aBMD and BMC compared with the low-stable trajectory in both sexes, in both crude and adjusted models (Tables 3 and 4). These associations were observed across skeletal sites, indicating generally consistent differences in skeletal level according to BMI development during adolescence.

**Table 3:** Model-based mean differences in overall areal BMD (aBMD, g/cm^2^) across BMI trajectory groups, estimated from repeated measurements across adolescence and young adulthood, stratified by sex, the Fit Futures study.

	Skeletal site	BMI trajectory	Model 0	*p*	Model 1	*p*	Model 2	*p*
**Females**	Total body (g/cm^2^)	Low-stable (ref.)	0		0		0	
		Moderate-increasing	0.07 [0.05, 0.08]	**<.001**	0.06 [0.04, 0.07]	**<.001**	0.06 [0.05, 0.08]	**<.001**
		High-stable	0.10 [0.07, 0.12]	**<.001**	0.10 [0.07, 0.12]	**<.001**	0.10 [0.07, 0.17]	**<.001**
	Total hip (g/cm^2^)	Low-stable (ref.)	0		0		0	
		Moderate-increasing	0.08 [0.06, 0.10]	**<.001**	0.07 [0.04, 0.09]	**<.001**	0.08 [0.05, 0.10]	**<.001**
		High-stable	0.12 [0.09, 0.16]	**<.001**	0.13 [0.09, 0.17]	**<.001**	0.13 [0.08, 0.19]	**<.001**
	Femoral neck (g/cm^2^)	Low-stable (ref.)	0		0		0	
		Moderate-increasing	0.07 [0.05, 0.10]	**<.001**	0.07 [0.04, 0.09]	**<.001**	0.07 [0.05, 0.10]	**<.001**
		High-stable	0.11 [0.07, 0.15]	**<.001**	0.11 [0.07, 0.15]	**<.001**	0.12 [0.06, 0.17]	**<.001**
**Males**	Total body (g/cm^2^)	Low-stable (ref.)	0		0		0	
		Moderate-increasing	0.07 [0.05, 0.09]	**<.001**	0.07 [0.05, 0.09]	**<.001**	0.08 [0.06, 0.10]	**<.001**
		High-stable	0.11 [0.07, 0.15]	**<.001**	0.08 [0.04, 0.13]	**<.001**	0.10 [0.06, 0.15]	**<.001**
	Total hip (g/cm^2^)	Low-stable (ref.)	0		0		0	
		Moderate-increasing	0.07 [0.04, 0.09]	**<.001**	0.07 [0.04, 0.10]	**<.001**	0.08 [0.05, 0.11]	**<.001**
		High-stable	0.13 [0.07, 0.18]	**<.001**	0.06 [−0.01, 0.13]	.08	0.07 [−0.01, 0.13]	.09
	Femoral neck (g/cm^2^)	Low-stable (ref.)	0		0		0	
		Moderate-increasing	0.07 [0.05, 0.10]	**<.001**	0.07 [0.04, 0.10]	**<.001**	0.08 [0.05, 0.11]	**<.001**
		High-stable	0.12 [0.07, 0.18]	**<.001**	0.06 [−0.01, 0.13]	.08	0.07 [0.01, 0.14]	**.045**

Values are β coefficients with 95% CI derived from linear mixed-effects models. *p*-values in bold indicate statistical significance at *p* < 0.05. The low-stable BMI trajectory group is used as the reference category in all analyses. Model 0: Unadjusted model; Model 1: Adjusted for baseline age (FF1) and pubertal maturation; Model 2: Additionally adjusted for physical activity, tobacco use, alcohol consumption, serum vitamin D level, and hormonal contraceptive use in females. Number of participants in the analyses: females (Model 0 = 559 Model 1 = 550, Model 2 = 522) and males (Model 0 = 553, Model 1 = 397, Model 2 = 380).

**Table 4:** Model-based mean differences in overall BMC (g) across BMI trajectory groups, estimated from repeated measurements across adolescence and young adulthood, stratified by sex, the Fit Futures study.

	Skeletal site	BMI trajectory	Model 0	*p*	Model 1	*p*	Model 2	*p*
**Females**	Total body (g)	Low-stable (ref.)	0		0		0	
		Moderate-increasing	326.5 [262.9, 390.1]	**<.001**	326.7 [258.1, 395.1]	**<.001**	341.2 [271.6410.8]	**<.001**
		High-stable	517.5 [406.9, 628.0]	**<.001**	546.6 [430.0, 395.1]	**<.001**	578.1 [461.8, 694.4]	**<.001**
	Total hip (g)	Low-stable (ref.)	0		0		0	
		Moderate-increasing	3.12 [2.24, 3.99]	**<.001**	3.06 [2.08, 4.05]	**<.001**	3.26 [2.32 4.19]	**<.001**
		High-stable	5.73 [4.21, 7.25]	**<.001**	6.24 [4.57, 7.91]	**<.001**	5.76 [4.21, 7.32]	**<.001**
	Femoral neck (g)	Low-stable (ref.)	0		0		0	
		Moderate-increasing	0.51 [0.39, 0.64]	**<.001**	0.50 [0.36, 0.64]	**<.001**	0.53 [0.39, 0.66]	**<.001**
		High-stable	0.74 [0.52, 0.96]	**<.001**	0.81 [0.57, 1.05]	**<.001**	0.78 [0.56, 1.01]	**<.001**
**Males**	Total body (g)	Low-stable (ref.)	0		0		0	
		Moderate-increasing	376.1 [300.0, 452.2]	**<.001**	373.3 [286.0, 460.4]	**<.001**	407.6 [320.0, 495.2]	**<.001**
		High-stable	584.9 [428.1, 741.6]	**<.001**	481.9 [286.5, 677.4]	**<.001**	513.2 [315.8, 710.7]	**<.001**
	Total hip (g)	Low-stable (ref.)	0		0		0	
		Moderate-increasing	3.36 [2.20, 4.51]	**<.001**	3.61 [2.23, 4.98]	**<.001**	3.93 [2.61, 5.26]	**<.001**
		High-stable	6.40 [4.03, 8.77]	**<.001**	3.82 [0.74, 6.91]	**.02**	4.47 [1.50, 7.45]	**.003**
	Femoral neck (g)	Low-stable (ref.)	0		0		0	
		Moderate-increasing	0.51 [0.34, 0.68]	**<.001**	0.55 [0.35 0.75]	**<.001**	0.62 [0.43, 0.81]	**<.001**
		High-stable	0.81 [0.46, 1.16]	**<.001**	0.39 [−0.06, 0.84]	.09	0.48 [0.05, 0.91]	**.032**

Values are β coefficients with 95% CI derived from linear mixed-effects models. *p*-values in bold indicate statistical significance at *p* < 0.05. The low-stable BMI trajectory group is used as the reference category in all analyses. Model 0: Unadjusted model; Model 1: Adjusted for baseline age (FF1) and pubertal maturation; Model 2: Additionally adjusted for physical activity, tobacco use, alcohol consumption, serum vitamin D level, and hormonal contraceptive use in females. Number of participants in the analyses: females (Model 0 = 559 Model 1 = 550, Model 2 = 522) and males (Model 0 = 553, Model 1 = 397, Model 2 = 380).

Among females, fully adjusted models demonstrated a clear gradient across BMI trajectories. Compared with the low-stable trajectory, the moderate-increasing trajectory was associated with higher aBMD at the TB (β = .06 g/cm^2^ [0.05, 0.08]), TH (β = .08 [0.05, 0.10]), and FN (β = .07 [0.05, 0.10]). Associations were consistently stronger in the high-stable trajectory, with β coefficients of 0.10 [0.07, 0.17], 0.13 [0.08, 0.19], and 0.12 [0.06, 0.17], respectively (Table 3). A similar gradient was observed for BMC across all skeletal sites (Table 4).

Among males, the moderate-increasing trajectory was associated with higher aBMD at the TB (β = .08 g/cm^2^ [0.06, 0.10]), TH (β = .08 [0.05, 0.11]), and FN (β = .08 [0.05, 0.11]) compared with the low-stable trajectory. The high-stable trajectory showed the strongest association for TB aBMD (β = .10 [0.06, 0.15]), whereas associations at the TH (β = .07 [−0.01, 0.13]) and FN (β = .07 [0.01, 0.14]) were slightly weaker than those observed for the moderate-increasing trajectory (Table 3). A similar pattern was observed for BMC, although the high-stable trajectory showed the largest estimates at the TB and TH, whereas the moderate-increasing trajectory showed the largest estimate at the FN (Table 4).

### Longitudinal changes in bone outcomes across BMI trajectories

**Figure 3:**
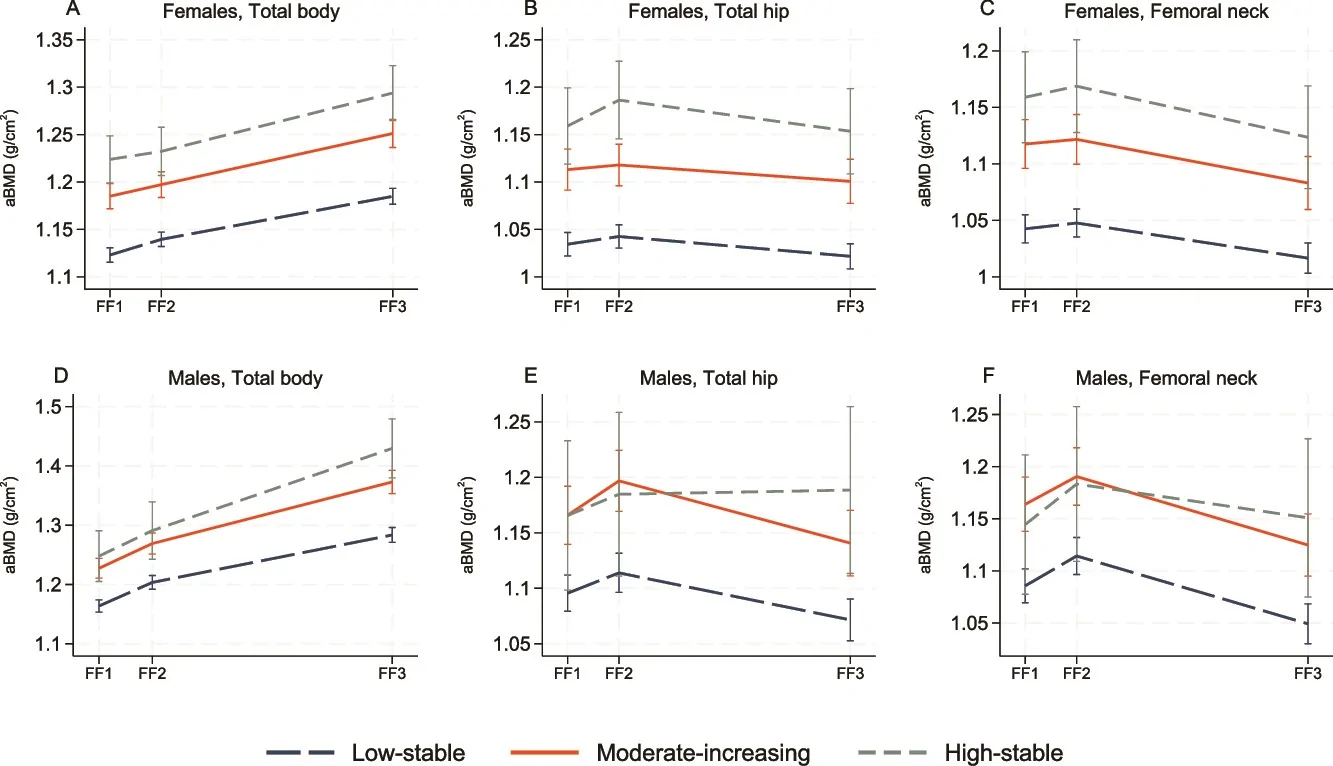
Model-estimated mean areal BMD (aBMD) across BMI trajectories from adolescence to young adulthood in females and males. Panels show total body (TB) (A), TH (B), and FN (C) aBMD in females, and TB (D), TH (E), and FN (F) aBMD in males. Estimates are presented for the low-stable, moderate-increasing, and high-stable BMI trajectory groups at FF1, FF2, and FF3. Error bars represent 95% CI. Models were adjusted for baseline age, pubertal maturation, physical activity, tobacco use, alcohol consumption, and vitamin D status, with additional adjustment for hormonal contraceptive use in females. FF1, Fit Futures 1 (2010-2011); FF2, Fit Futures 2 (2012-2013); FF3, Fit Futures 3 (2021-2022).

**Figure 4:**
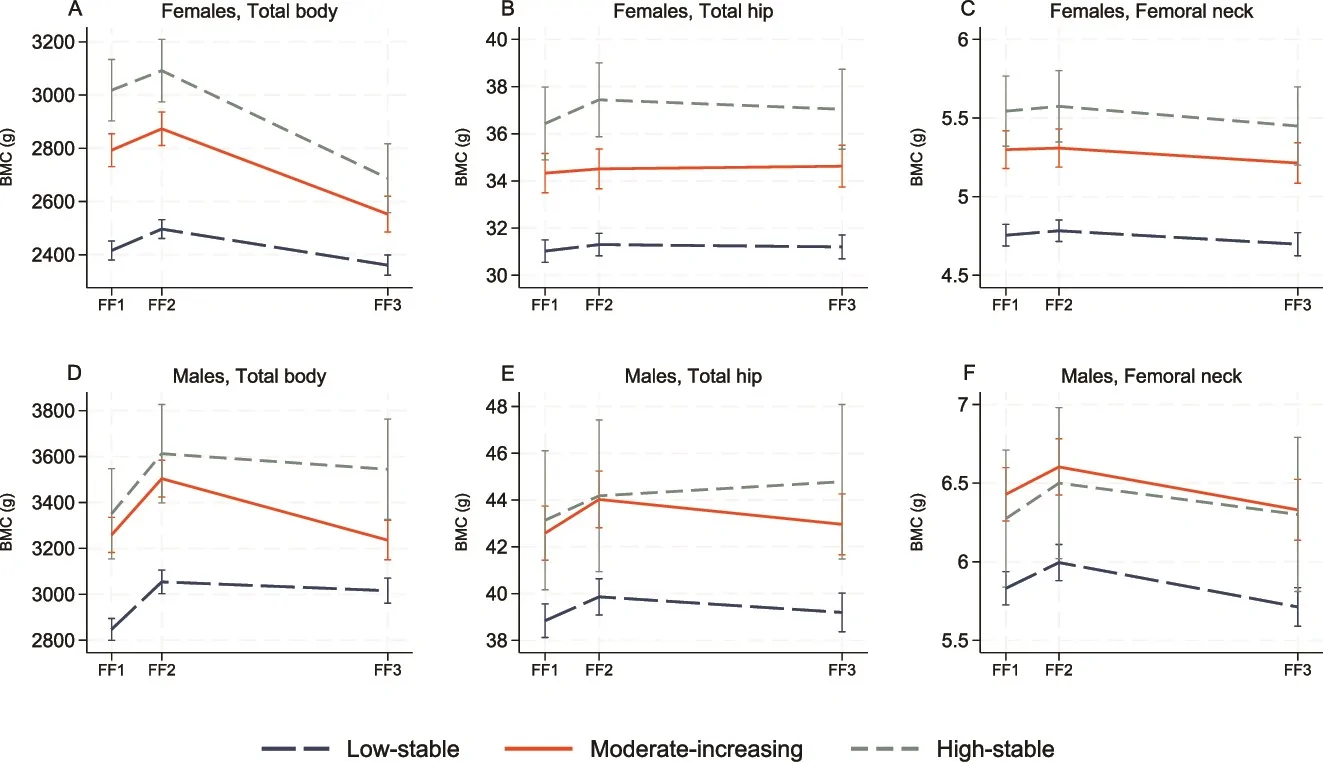
Model-estimated mean BMC across BMI trajectories from adolescence to young adulthood in females and males. Panels show total body (TB) (A), TH (B), and FN (C) BMC in females, and TB (D), TH (E), and FN (F) BMC in males. Estimates are presented for the low-stable, moderate-increasing, and high-stable BMI trajectory groups at FF1, FF2, and FF3. Error bars represent 95% CI. Models were adjusted for baseline age, pubertal maturation, physical activity, tobacco use, alcohol consumption, and vitamin D status, with additional adjustment for hormonal contraceptive use in females. FF1, Fit Futures 1 (2010-2011); FF2, Fit Futures 2 (2012-2013); FF3, Fit Futures 3 (2021-2022).

When time was incorporated into the models, adjusted mean aBMD and BMC remained consistently higher in the moderate-increasing and high-stable BMI trajectories than in the low-stable trajectory throughout follow-up (Figures 3 and 4; Tables S2 and S3). Formal BMI trajectory × timepoint interaction tests demonstrated that longitudinal changes were largely parallel across BMI trajectories at the TH and FN. Statistically significant trajectory × time interactions were limited to TB outcomes, including TB aBMD and BMC in males and TB BMC in females (Tables S4 and S5).

Among females, model-based estimates indicated increases in TB aBMD from FF1 to FF3 across all BMI trajectories, ranging from 0.07 g/cm^2^ [0.06, 0.08] in the low-stable trajectory and 0.06 [0.05, 0.08] in the moderate-increasing trajectory to 0.11 [0.07, 0.15] in the high-stable trajectory. In contrast, estimated changes at the TH and FN were small, ranging from −0.02 to 0.04 g/cm^2^ across trajectories (Table 5; Figure 3A-C). For BMC, TB values increased from FF1 to FF2 before declining by FF3, resulting in the only statistically significant BMI trajectory × time interaction observed among females, whereas no statistically significant interactions were observed for the TH or FN (Table 6; Figure 4A-C; Table S4).

**Table 5:** Adjusted within-group changes in areal BMD (aBMD, g/cm^2^) from FF1 to FF2 and FF3 by BMI trajectory group, stratified by sex, the Fit Futures study.

	Skeletal site	BMI trajectory	FF1 (adjusted mean)	ΔFF1 → FF2	*p*	ΔFF1 → FF3	*p*
**Females**	Total body (g/cm^2^)	Low-stable	0.79 [0.56, 1.01]	0.02 [0.01, 0.02]	**<.001**	0.07 [0.06, 0.08]	**<.001**
		Moderate-increasing	0.85 [0.62, 1.08]	0.02 [0.01, 0.03]	**.025**	0.06 [0.05, 0.08]	**<.001**
		High-Stable	0.89 [0.66, 1.12]	0.02 [−0.02, 0.05]	.33	0.11 [0.07, 0.15]	**<.001**
	Total hip (g/cm^2^)	Low-stable	0.87 [0.45, 1.24]	0.01 [−0.01, 0.02]	.27	−0.01 [−0.02, 0.01]	.16
		Moderate-increasing	0.92 [0.53, 1.31]	−0.01 [−0.02, 0.02]	.95	−0.01 [−0.03, 0.02]	.73
		High-Stable	0.95 [0.55, 1.35]	0.05 [0.01, 0.09]	**.017**	0.04 [−0.02, 0.09]	.16
	Femoral neck (g/cm^2^)	Low-stable	0.83 [0.45, 1.22]	0.01 [−0.01, 0.01]	.82	−0.02 [−0.03, −0.01]	**.005**
		Moderate-increasing	0.90 [0.52, 1.29]	0.01 [−0.02, 0.02]	.67	−0.02 [−0.04, 0.01]	.13
		High-Stable	0.94 [0.55, 1.34]	0.01 [−0.04, 0.05]	.94	−0.01 [−0.06, 0.06]	.98
**Males**	Total body (g/cm^2^)	Low-stable	1.06 [0.82, 1.30]	0.06 [0.04, 0.08]	**<.001**	0.12 [0.11, 0.13]	**<.001**
		Moderate-increasing	1.12 [0.88, 1.37]	0.04 [0.03, 0.05]	**<.001**	0.15 [0.12, 0.16]	**<.001**
		High-stable	1.14 [0.90, 1.39]	0.04 [0.01, 0.10]	**.016**	0.18 [0.16, 0.22]	**<.001**
	Total hip (g/cm^2^)	Low-stable	1.19 [0.80, 1.58]	0.02 [0.01, 0.03]	**.002**	−0.02 [−0.04, −0.01]	**<.001**
		Moderate-increasing	1.26 [0.87, 1.65]	0.03 [0.01, 0.05]	**<.001**	−0.03 [−0.05, −0.01]	**.013**
		High-stable	1.26 [0.87, 1.66]	0.02 [−0.03, 0.07]	.41	0.02 [−0.03, 0.07]	.35
	Femoral neck (g/cm^2^)	Low-stable	1.05 [0.66, 1.43]	0.02 [−0.03, 0.07]	**<.001**	0.02 [−0.03, 0.07]	**<.001**
		Moderate-increasing	1.12 [0.74, 1.51]	0.03 [0.02, 0.04]	**.005**	−0.04 [−0.05, −0.02]	**<.001**
		High-stable	1.10 [0.71, 1.50]	0.03 [0.01, 0.05]	.12	−0.04 [−0,06, −0.02]	.81

Values are presented as regression coefficients (β) with 95% CI. *p*-values in bold indicate statistical significance at *p* < 0.05. Estimates represent adjusted within-group changes in aBMD between FF1 and FF2, and between FF1 and FF3. Models were fitted using linear mixed-effects regression with random intercepts and adjusted for baseline age (FF1), pubertal maturation, physical activity, tobacco use, alcohol consumption, serum vitamin D status, and hormonal contraceptive use in females. FF1 was specified as the reference time point in all models.

**Table 6:** Adjusted within-group changes in BMC (g) from FF1 to FF2 and FF3 by BMI trajectory group, stratified by sex, the Fit Futures study.

	Skeletal site	BMI trajectory	FF1 (adjusted mean)	ΔFF1 → FF2	*p*	ΔFF1 → FF3	*p*
**Females**	Total body (g)	Low-stable	1374.3 [403.6, 2345]	78.4 [48.8, 108.0]	**<.001**	−29.6 [−65.6, 6.4]	.11
		Moderate-increasing	1759.5 [790.1, 2729]	100.1 [48.4, 153.6]	**<.001**	−216.2 [−280.1, −152.3]	**<.001**
		High-Stable	1962.1 [967.8, 2956.5]	86.2 [−35.7, 208.0]	.17	−229.1 [−387.2, −70.8]	**.005**
	Total hip (g)	Low-stable	21.69 [6.96, 36.43]	0.13 [−0.23, 0.48]	.49	0.34 [−0.10, .078]	.13
		Moderate-increasing	25.07 [10.36, 39.78]	−0.02 [−0.66, 0.61]	.94	0.62 [−0.16, 1.40]	.12
		High-Stable	26.59 [11.52, 41.67]	2.10 [0.61, 3.59]	**.006**	2.51 [0.55, 4.47]	**.012**
	Femoral neck (g)	Low-stable	3.16 [1.09, 5.23]	0.01 [−0.05, 0.06]	.75	−0.02 [−0.08, 0.05]	.65
		Moderate-increasing	3.73 [1.66, 5.79]	−0.01 [−0.10, 0.09]	.94	−0.01 [−0.12, 0.11]	.95
		High-Stable	4.07 [1.96, 6.19]	−0.07 [−0.29, 0.16]	.57	−0.01 [−0.31, 0.29]	.94
**Males**	Total body (g)	Low-stable	2418.6 [1280.6, 3556.6]	206.9 [173.6, 240.2]	**<.001**	168.6 [130.9, 206.2]	**<.001**
		Moderate-increasing	2829.7 [1691.1, 3968.3]	245.2 [196.4, 294.1]	**<.001**	−22.4 [−78.6, 33.7]	.43
		High-stable	2922 [1762.4, 4081.5]	261.7 [131.4, 392.0]	**<.001**	193.7 [56.7, 330.7]	**.006**
	Total hip (g)	Low-stable	37.92 [20.70, 55.15]	1.02 [0.51, 1.52]	**<.001**	0.35 [−0.22, 0.92]	.23
		Moderate-increasing	41.67 [24.43, 58.90]	1.44 [0.70, 2.18]	**<.001**	0.38 [−0.48, 1.24]	.39
		High-stable	42.22 [24.67, 59.77]	1.05 [−0.93, 3.02]	.30	1.65 [−0.43, 3.72]	.12
	Femoral neck (g)	Low-stable	5.52 [3.01, 8.04]	0.16 [0.09, 0.24]	**<.001**	−0.12 [−0.21, −0.03]	**.009**
		Moderate-increasing	6.12 [3.60, 8.63]	0.17 [0.06, 0.29]	**.003**	−0.10 [−0.23, 0.04]	.15
		High-stable	5.96 [3.40, 8.53]	0.23 [−0.08, 0.53]	.15	0.03 [−0.30, 0.35]	.87

Values are presented as regression coefficients (β) with 95% CI. *p*-values in bold indicate statistical significance at *p* < 0.05. Estimates represent adjusted within-group changes in BMC between FF1 and FF2, and between FF1 and FF3. Models were fitted using linear mixed-effects regression with random intercepts and adjusted for baseline age (FF1), pubertal maturation, physical activity, tobacco use, alcohol consumption, serum vitamin D status, and hormonal contraceptive use in females. FF1 was specified as the reference time point in all models.

Among males, model-based estimates indicated increases in TB aBMD from FF1 to FF3 across all BMI trajectories, ranging from 0.12 g/cm^2^ [0.11, 0.13] in the low-stable trajectory and 0.15 [0.12, 0.16] in the moderate-increasing trajectory to 0.18 [0.16, 0.22] in the high-stable trajectory. Estimated changes at the TH and FN were small across trajectories, ranging from −0.04 to 0.02 g/cm^2^ (Table 5; Figure 3D-F). Consistent with these findings, a statistically significant BMI trajectory × time interaction was observed for TB aBMD only, whereas no statistically significant interactions were observed for the TH or FN (Table S5). For BMC, TB values increased from FF1 to FF2 and remained relatively stable thereafter, whereas BMC at the TH and FN remained largely stable throughout follow-up. Accordingly, a significant BMI trajectory × time interaction was observed only for TB BMC, whereas no significant interactions were observed for the TH or FN (Table 6; Figure 4D-F; Table S5).

Overall, the results showed that differential longitudinal change was confined to TB outcomes.

## Discussion

The principal finding of this study was that BMI trajectories established during adolescence were associated with persistent differences in skeletal level rather than differential skeletal accrual into young adulthood. Participants in the low-stable trajectory consistently exhibited the lowest aBMD and BMC throughout follow-up, whereas those in the moderate-increasing and high-stable trajectories consistently exhibited higher values in both females and males. Despite sustained increases in BMI in the moderate-increasing trajectory, longitudinal skeletal gains were generally comparable with those observed in the high-stable trajectory.

### Sex-specific longitudinal patterns in bone outcomes

Longitudinal patterns differed between females and males, consistent with established sex differences in skeletal maturation. Females showed only modest longitudinal changes in bone outcomes throughout follow-up, reflecting skeletal consolidation after late adolescence and the earlier attainment of PBM. This pattern is consistent with longitudinal evidence demonstrating that bone accrual declines rapidly after menarche, with little additional bone gain within a few years thereafter.^2^ Despite these limited changes, BMI trajectory groups remained clearly separated across all skeletal sites, suggesting that higher BMI trajectories were primarily associated with higher attained bone mass rather than greater skeletal accrual over time.

In contrast, males demonstrated more prolonged skeletal development, consistent with continued periosteal expansion and later attainment of PBM.^2^ However, despite this extended period of skeletal maturation, longitudinal changes were generally comparable across BMI trajectory groups, suggesting that higher BMI trajectories likewise reflected differences in attained skeletal level rather than accelerated skeletal accrual.

These findings are consistent with previous observations from the Fit Futures cohort and other longitudinal studies reporting earlier skeletal maturation in females and more prolonged skeletal development in males.^4^^,^^20^ Our findings further suggest that these sex-specific developmental patterns are preserved across BMI trajectory groups.

### Site-specific patterns of skeletal development

Longitudinal changes also differed by skeletal site. In both sexes, the greatest changes were observed for TB outcomes, whereas changes at the TH and FN were comparatively modest. This site-specific pattern was particularly evident in males, in whom TB aBMD and BMC continued to increase throughout follow-up, while aBMD at the TH and FN stabilized or declined after late adolescence. Among females, changes in bone outcomes were modest across all skeletal sites, although TB outcomes continued to show slightly greater variation than the regional skeletal measurements.

Corresponding BMC findings followed the same pattern, with greater longitudinal change at the TB than at the TH and FN.

These findings are biologically plausible, as previous longitudinal studies have demonstrated that the timing of PBM differs across skeletal sites, with PBM being attained earlier at the FN and TH than at the TB, reflecting site-specific patterns of skeletal maturation.^3^^,^^39^ Furthermore, whole-body DXA reflects the combined development of multiple skeletal regions with distinct growth trajectories and maturation rates. Consequently, whole-body measurements may continue to capture ongoing skeletal changes even after regional skeletal sites have largely reached PBM.

Together, these findings highlight that the relationship between long-term BMI trajectories and bone development should be interpreted in the context of skeletal sites.

### High BMI trajectories: protective but not progressive

Higher BMI trajectories were consistently associated with higher aBMD and BMC throughout follow-up, but higher BMI did not necessarily translate into proportionally greater skeletal accrual across all skeletal sites. Although participants in the high-stable trajectory generally exhibited the highest bone values by young adulthood (FF3), greater longitudinal gains were primarily observed for TB outcomes. In contrast, longitudinal changes at weight-bearing sites were modest and largely comparable between the moderate-increasing and high-stable trajectories. This pattern was particularly evident among males, where the moderate-increasing trajectory demonstrated slightly higher aBMD at the TH and FN during adolescence despite a lower BMI.

Previous studies have demonstrated positive associations between body size and bone mass during growth and adolescence.^11^^,^^12^ In this context, a persistently high BMI appears to represent a favorable skeletal profile characterized by consistently higher bone mass throughout follow-up. However, our findings extend this evidence by showing that persistently high BMI was associated with higher attained bone mass without consistently influencing the subsequent pattern of skeletal development. Thus, while higher BMI appeared to be protective in terms of skeletal level, it was not associated with proportionally greater longitudinal gains across all skeletal sites.

According to the mechanostat theory, increases in body mass increase habitual mechanical loading, thereby stimulating bone formation and skeletal adaptation.^1^^,^^2^^,^^8^^,^^40^ Such loading may be particularly important during periods of rapid skeletal growth, when the skeleton is most responsive to mechanical stimuli. However, once skeletal maturity is approached, the potential for additional bone accrual becomes more limited. Consequently, maintaining a persistently high BMI may contribute more to preserving an already higher skeletal level than to promoting continued bone accrual.

Collectively, these findings suggest that the primary skeletal benefit of higher BMI lies in achieving and maintaining a higher bone mass rather than accelerating subsequent skeletal development. Distinguishing between attained skeletal level and skeletal accrual may therefore improve our understanding of how long-term BMI development influences PBM and future skeletal health.

### Increasing BMI: not necessarily greater bone accrual

A notable finding of the present study was that increasing BMI during adolescence and into adulthood, did not consistently translate into greater skeletal accrual. Participants in the moderate-increasing trajectory experienced sustained increases in BMI throughout follow-up, yet longitudinal gains in aBMD and BMC were generally comparable to those observed in the high-stable trajectory. These findings suggest that increasing BMI alone does not necessarily translate into greater skeletal accrual during adolescence and young adulthood.

This interpretation is consistent with longitudinal evidence suggesting that skeletal accrual during adolescence is influenced by biological factors beyond BMI alone. In a prospective study of adolescents, Luiz-de-Marco et al. reported that gains in lean soft tissue explained a substantial proportion of lower-limb aBMD accrual, whereas changes in fat mass were not independently associated with aBMD gain after adjustment for body weight and maturational timing.^41^ Similarly, accumulating evidence indicates that lean mass is a major determinant of skeletal adaptation, particularly at weight-bearing sites, whereas adiposity may exert more complex or even adverse effects on bone depending on metabolic and mechanical context.^42^

Taken together, these observations suggest that BMI alone does not fully capture the biological processes underlying skeletal adaptation during growth. Higher BMI trajectories were primarily associated with higher attained skeletal levels rather than greater longitudinal skeletal accrual, supporting the interpretation of BMI trajectories as markers of long-term body size rather than predictors of continued skeletal accrual. Descriptive body composition data in our study further demonstrated that higher BMI trajectories were characterized by higher levels of both lean and fat mass throughout follow-up. This distinction may explain why the moderate-increasing trajectory exhibited a favorable skeletal profile relative to the low-stable trajectory without consistently demonstrating greater skeletal accrual or attaining the skeletal levels observed in the high-stable trajectory.

### Persistently low BMI: a skeletal risk profile

A persistently low BMI trajectory emerged as the least favorable skeletal profile in the present study. Although this trajectory consisted predominantly of individuals within the normal BMI range rather than underweight participants, it was consistently associated with lower aBMD and BMC throughout follow-up. Importantly, differences between BMI trajectories remained largely unchanged over time, indicating that participants in the low-stable trajectory attained a lower skeletal level during adolescence that persisted into young adulthood, with little indication of skeletal catch-up.

Although females and males displayed different patterns of skeletal maturation, participants in the low-stable trajectory remained consistently below the other BMI trajectories throughout follow-up, despite increasing levels of both fat and lean mass within the group. Females showed only modest longitudinal changes during late adolescence, whereas males continued to accrue bone mass over a longer period. However, the more prolonged skeletal development observed in males was insufficient to eliminate the differences established during adolescence, suggesting that continued skeletal maturation alone does not compensate for an initially lower skeletal level.

These findings are consistent with previous longitudinal studies demonstrating that body size during adolescence tracks into adulthood and influences PBM.^11^^,^^43^ Previous analyses from the Fit Futures cohort similarly reported lower bone mass among adolescents with lower BMI, particularly among boys, and demonstrated that underweight boys remained disadvantaged during the first 2 yr of follow-up.^15^ The present study extends these findings by demonstrating that these differences persist throughout the transition into young adulthood.

These findings suggest that individuals following a persistently low BMI trajectory establish a lower skeletal reserve during adolescence that is not fully recovered by young adulthood, reinforcing adolescence as a critical period for PBM acquisition. Consequently, inadequate bone accrual during this period may have lasting implications for skeletal health and increase the risk of osteoporosis and fractures later in life.^1–3^^,^^5^^,^^11^

Collectively, our findings suggest that persistently low BMI represents a skeletal risk profile characterized by lower attained bone mass rather than impaired skeletal development per se. Rather than indicating an inability to continue accruing bone, the present findings indicate that individuals in the low-stable trajectory established a lower skeletal reserve during adolescence that was not fully recovered by young adulthood. These findings underscore the importance of optimizing bone accrual during adolescence to maximize PBM and promote lifelong skeletal health.

### Methodological considerations

A major strength of this study is the longitudinal, population-based design with repeated DXA assessments spanning the transition from adolescence into young adulthood, a critical period for skeletal development.^1^ Group-based BMI trajectory modeling enabled characterization of long-term BMI development rather than relying on single BMI measurements or short-term changes, allowing differentiation between attained skeletal level and longitudinal skeletal accrual across clinically meaningful BMI trajectories. Inclusion of both females and males and multiple skeletal sites enabled sex- and site-specific interpretation of skeletal development, while adjustment for pubertal timing and relevant lifestyle-related covariates strengthened the internal validity of the findings.^1^^,^^30^

Some limitations should be acknowledged. First, BMI was used as a proxy for body size but does not distinguish between fat and lean mass.^42^ Consequently, BMI trajectories should be interpreted as markers of long-term body size rather than direct measures of the biological processes driving skeletal adaptation. In addition, limited group sizes across BMI trajectories required pooling of individuals from different BMI categories. Consequently, persistent underweight could not be identified as a separate trajectory, and the high-stable trajectory group was relatively small, reducing statistical precision.

Second, although DXA is widely used in epidemiological studies, aBMD is a 2-dimensional measure influenced by bone size, and changes in bone geometry or volumetric density cannot be assessed, which is particularly relevant during adolescence and young adulthood.^40^^,^^44^ A study-specific in vivo precision assessment was not performed for the Fit Futures study. Consequently, the precision error under the specific acquisition conditions of this study is unknown. However, previous studies have reported acceptable precision for DXA-derived BMD measurements using the same scanner.^26^ DXA measurement error, including positioning- and acquisition-related variability, may therefore have contributed to the observed variability in BMD changes. To the extent that such errors were non-differential across BMI trajectory groups, they would be expected to reduce precision rather than systematically bias the estimated group-level changes.

Residual confounding cannot be excluded. Dietary factors, including energy, calcium, and protein intake, were unavailable, socioeconomic conditions were not assessed, and attrition during follow-up may have introduced selection bias.

Finally, pubertal maturation was assessed using different sex-specific measures. Age at menarche reflects pubertal timing, whereas PDS reflects PDS at the time of assessment. Although both measures were included to account for maturation within each sex, they capture different aspects of biological development and are therefore not directly comparable. Furthermore, a substantial proportion of boys had missing PDS data. Sensitivity analyses suggested only minor differences between boys with and without available PDS data, although those with PDS data were slightly older and had marginally higher TB aBMD. Consequently, some residual confounding related to pubertal maturation cannot be excluded. A harmonized measure such as peak height velocity might have provided more comparable adjustment across sexes, but these data were unavailable in Fit Futures. Nevertheless, the use of linear mixed-effects models allowed inclusion of participants with incomplete follow-up, mitigating bias and preserving statistical power.^38^

### Conclusion

In this longitudinal population-based study, sustained BMI trajectories during adolescence were associated with distinct skeletal profiles from adolescence into young adulthood. Higher BMI trajectories were consistently associated with higher aBMD and BMC levels, whereas the low-stable trajectory exhibited the least favorable skeletal profile throughout follow-up. Importantly, these differences primarily reflected attained skeletal level rather than greater longitudinal skeletal accrual, indicating that increasing BMI during adolescence was not associated with proportionally greater gains in bone outcomes over time.

These findings suggest that BMI-related differences in bone health are established during adolescence and largely maintained into young adulthood, highlighting adolescence as a critical period for the attainment of PBM. The absence of proportionally greater skeletal gains despite increasing BMI indicates that long-term BMI trajectories primarily influence the skeletal level attained by early adulthood rather than longitudinal skeletal accrual, emphasizing the importance of healthy growth patterns during adolescence for lifelong skeletal health.

## Supplementary Material

Supplementary_material__ziag143

## Data Availability

The data supporting the findings of this study are not publicly available due to restrictions related to participant consent and data protection. Access may be requested from the Fit Futures study at UiT-The Arctic University of Norway, Department of Community Medicine (fitfutures@uit.no), subject to approval by the data owner and relevant regulatory authorities.

## References

[1] Weaver CM, Gordon CM, Janz KF, et al. The National Osteoporosis Foundation’s position statement on peak bone mass development and lifestyle factors: a systematic review and implementation recommendations. Osteoporos Int. 2016;27(4):1281–1386. 10.1007/s00198-015-3440-3 26856587 PMC4791473

[2] Chevalley T, Rizzoli R. Acquisition of peak bone mass. Best Pract Res Clin Endocrinol Metab. 2022;36(2):101616. 10.1016/j.beem.2022.101616 35125324

[3] Berger C, Goltzman D, Langsetmo L, et al. Peak bone mass from longitudinal data: implications for the prevalence, pathophysiology, and diagnosis of osteoporosis. J Bone Miner Res. 2010;25(9):1948–1957. 10.1002/jbmr.95 20499378 PMC5101070

[4] Bonjour J-P, Chevalley T. Pubertal timing, bone acquisition, and risk of fracture throughout life. Endocr Rev. 2014;35(5):820–847. 10.1210/er.2014-1007 25153348

[5] Hereford T, Kellish A, Samora JB, Reid NL. Understanding the importance of peak bone mass. J Pediatr Soc North Am. 2024;7(5):100031. 10.1016/j.jposna.2024.100031 40433296 PMC12088333

[6] McCormack SE, Cousminer DL, Chesi A, et al. Association between linear growth and bone accrual in a diverse cohort of children and adolescents. JAMA Pediatr. 2017;171(9):e171769. 10.1001/jamapediatrics.2017.1769 28672287 PMC5632753

[7] Bierhals IO, dos Santos VJ, Bielemann RM, et al. Associations between body mass index, body composition and bone density in young adults: findings from a southern Brazilian cohort. BMC Musculoskelet Disord. 2019;20(1):322–310. 10.1186/s12891-019-2656-3 31288773 PMC6617655

[8] Meslier QA, Shefelbine SJ. Using finite element modeling in bone mechanoadaptation. Curr Osteoporos Rep. 2023;21(2):105–116. 10.1007/s11914-023-00776-9 36808071 PMC10105683

[9] Farella I, Chiarito M, Vitale R, D’Amato G, Faienza MF. The “burden” of childhood obesity on bone health: a look at prevention and treatment. Nutrients. 2025;17(3):491. 10.3390/nu17030491 39940349 PMC11821239

[10] Wu Y, Li D, Vermund SH. Advantages and limitations of the body mass index (BMI) to assess adult obesity. Int J Environ Res Public Health. 2024;21(6):757. 10.3390/ijerph21060757 38929003 PMC11204233

[11] Simchoni M, Landau R, Derazne E, et al. Adolescent body mass index, weight trajectories to adulthood, and osteoporosis risk. JAMA Netw Open. 2025;8(8):e2525079. 10.1001/jamanetworkopen.2025.25079 40758350 PMC12322795

[12] Qiao D, Li Y, Liu X, et al. Association of obesity with bone mineral density and osteoporosis in adults: a systematic review and meta-analysis. Public Health. 2020;180:22–28. 10.1016/j.puhe.2019.11.001 31837611

[13] Greco EA, Fornari R, Rossi F, et al. Is obesity protective for osteoporosis? Evaluation of bone mineral density in individuals with high body mass index. Int J Clin Pract. 2010;64(6):817–820. 10.1111/j.1742-1241.2009.02301.x 20518955

[14] Rinonapoli G, Pace V, Ruggiero C, et al. Obesity and bone: a complex relationship. Int J Mol Sci. 2021;22(24):13662. 10.3390/ijms222413662 34948466 PMC8706946

[15] Nilsen OA, Ahmed LA, Winther A, et al. Body weight and body mass index influence bone mineral density in late adolescence in a two-year follow-up study. The Tromsø study: fit futures. JBMR Plus. 2019;3(9):e10195. 10.1002/jbm4.10195 31667452 PMC6808229

[16] Gállego Suárez C, Singer BH, Gebremariam A, Lee JM, Singer K. The relationship between adiposity and bone density in U.S. children and adolescents. PLoS One. 2017;12(7):e0181587. 10.1371/journal.pone.0181587 28723934 PMC5517060

[17] Lorentzon M, Mellström D, Ohlsson C. Age of attainment of peak bone mass is site specific in Swedish men—the GOOD study. J Bone Miner Res. 2005;20(7):1223–1227. 10.1359/JBMR.050306 15940376

[18] Lemanowicz-Kustra A, Brzeziński M, Dettlaff-Dunowska M, et al. Body weight changes during childhood and predictors of excessive body weight in adolescence—a longitudinal analysis. Nutrients. 2024;16(24):4397. 10.3390/nu16244397 39771018 PMC11676939

[19] Bermudez B, Ishii T, Wu Y-H, Carpenter RD, Sherk VD. Energy balance and bone health: a nutrient availability perspective. Curr Osteoporos Rep. 2023;21(1):77–84. 10.1007/s11914-022-00765-4 36542294

[20] Sagelv EH, Emaus N, Evensen E, et al. Acquisition of peak bone mass in a Norwegian youth cohort: longitudinal findings from the Fit Futures study 2010–2022. Arch Osteoporos. 2024;19(1):58. 10.1007/s11657-024-01414-2 38960953 PMC11222189

[21] Nagin DS, Jones BL, Elmer J. Recent advances in group-based trajectory modeling for clinical research. Annu Rev Clin Psychol. 2024;20(1):285–305. 10.1146/annurev-clinpsy-081122-012416 38382118

[22] Evensen E, Skeie G, Wilsgaard T, et al. How is adolescent bone mass and density influenced by early life body size and growth? The Tromsø study: Fit Futures—a longitudinal cohort study from Norway. JBMR Plus. 2018;2(5):268–280. 10.1002/jbm4.10049 30283908 PMC6139726

[23] Regulation on Population-Based Health Research . FOR-2009-02-20-219. Norwegian Ministry of Health and Care Services; 2009.

[24] Health Research Act . LOV-2008-06-20-44. Norwegian Ministry of Health and Care Services; 2008.

[25] Personal Data Act . LOV-2018-06-15-38. Norwegian Ministry of Justice and Public Security; 2018.

[26] Omsland TK, Emaus N, Gjesdal CG, et al. In vivo and In vitro comparison of densitometers in the NOREPOS study. J Clin Densitom. 2008;11(2):276–282. 10.1016/j.jocd.2007.10.001 18158262

[27] GE Healthcare . GE Healthcare Lunar enCORE-Based X-Ray Bone Densitometer User Manual, Revision 5, LU43616EN. GE Healthcare; 2010.

[28] World Health Organization . Obesity: Preventing and Managing the Global Epidemic: Report of a WHO Consultation. World Health Organization; 2000: Report No.: 894. 11234459

[29] Cole TJ, Lobstein T. Extended international (IOTF) body mass index cut-offs for thinness, overweight and obesity. Pediatr Obes. 2012;7(4):284–294. 10.1111/j.2047-6310.2012.00064.x 22715120

[30] Gordon CM, Zemel BS, Wren TAL, et al. The determinants of peak bone mass. J Pediatr. 2017;180:261–269. 10.1016/j.jpeds.2016.09.056 27816219

[31] Petersen AC, Crockett L, Richards M, Boxer A. A self-report measure of pubertal status: reliability, validity, and initial norms. J Youth Adolesc. 1988;17(2):117–133. 10.1007/BF01537962 24277579

[32] Öberg J, Jorde R, Almås B, et al. Vitamin D status during adolescence and the impact of lifestyle changes: 2 years’ follow-up from the fit futures study. J Clin Endocrinol Metab. 2024;109(3):e1029–e1039. 10.1210/clinem/dgad655 37955862 PMC10876399

[33] Ross AC, Taylor CL, Yaktine AL, Del Valle HB, Institute of M, Heather BDV, et al. Dietary Reference Intakes for Calcium and Vitamin D. 1st ed. National Academies Press; 2011. 21796828

[34] Grimby G, Börjesson M, Jonsdottir IH, Schnohr P, Thelle DS, Saltin B. The "Saltin-Grimby Physical Activity Level Scale" and its application to health research. Scand J Med Sci Sports. 2015;25(S4):119–125. 10.1111/sms.12611 26589125

[35] Leonard MB, Elmi A, Mostoufi-Moab S, et al. Effects of sex, race, and puberty on cortical bone and the functional muscle bone unit in children, adolescents, and young adults. J Clin Endocrinol Metab. 2010;95(4):1681–1689. 10.1210/jc.2009-1913 20157194 PMC2853999

[36] Jones BL, Nagin DS. A note on a Stata plugin for estimating group-based trajectory models. Sociol Methods Res. 2013;42(4):608–613. 10.1177/0049124113503141

[37] Nagin D. Group-Based Modeling of Development. Harvard University Press; 2005. 10.4159/9780674041318.

[38] Twisk JWR . Applied Mixed Model Analysis: a Practical Guide. Cambridge University Press; 2019, 10.1017/9781108635660.

[39] Baxter-Jones ADG, Faulkner RA, Forwood MR, Mirwald RL, Bailey DA. Bone mineral accrual from 8 to 30 years of age: an estimation of peak bone mass. J Bone Miner Res. 2011;26(8):1729–1739. 10.1002/jbmr.412 21520276

[40] Wasserman H, O'Donnell JM, Gordon CM. Use of dual energy X-ray absorptiometry in pediatric patients. Bone. 2017;104:84–90. 10.1016/j.bone.2016.12.008 27989544 PMC7055510

[41] Luiz-de-Marco R, Gobbo LA, Castoldi RC, et al. Impact of changes in fat mass and lean soft tissue on bone mineral density accrual in adolescents engaged in different sports: ABCD growth study. Arch Osteoporos. 2020;15(1):22. 10.1007/s11657-020-0707-x 32090287

[42] Nguyen HG, Pham MTD, Ho-Pham LT, Nguyen TV. Lean mass and peak bone mineral density. Osteoporos Sarcopenia. 2020;6(4):212–216. 10.1016/j.afos.2020.10.001 33426311 PMC7783218

[43] Wren TAL, Kalkwarf HJ, Zemel BS, et al. Longitudinal tracking of dual-energy X-ray absorptiometry bone measures over 6 years in children and adolescents: persistence of low bone mass to maturity. J Pediatr. 2014;164(6):1280–5.e2. 10.1016/j.jpeds.2013.12.040 24485819 PMC4035430

[44] Golding PH . Dual-energy X-ray absorptiometry (DXA) to measure bone mineral density (BMD) for diagnosis of osteoporosis - experimental data from artificial vertebrae confirms significant dependence on bone size. Bone Rep. 2022;17:101607. 10.1016/j.bonr.2022.101607 35937936 PMC9352459

