# Supplementary material for: Associations between BMI trajectories and young adult bone: a 10-yr follow-up from the Norwegian Fit Futures cohort
Source: JBMR Plus. 2026 Aug 25;10(10):ziag143. doi: 10.1093/jbmrpl/ziag143 (PMC13549439; doi:10.1093/jbmrpl/ziag143)
Supplement: Supplementary file 1 [file supplementary_material__ziag143.zip › Supplementary Table S5.docx]

**Supplementary Table S5** Interaction between BMI trajectory group and timepoint in males, The Fit Future study

| Outcome | χ² (4) | *p* (traj × time) |
| --- | --- | --- |
| Total body aBMD (g/cm^2^) | 16.83 | **0.0021** |
| Total hip aBMD (g/cm^2^) | 5.77 | 0.22 |
| Femoral neck aBMD (g/cm^2^) | 2.62 | 0.62 |
| Total body BMC (g) | 45.48 | **0.0000** |
| Total hip BMC (g) | 2.64 | 0.62 |
| Femoral neck BMC (g) | 0.77 | 0.94 |

*P-values are derived from Wald χ² tests of the joint BMI trajectory–by–timepoint interaction terms in linear mixed-effects models.* *A significant interaction indicates differential longitudinal change between BMI trajectory groups, whereas a non-significant interaction indicates parallel change over time.*
