# Supplementary material for: Associations between BMI trajectories and young adult bone: a 10-yr follow-up from the Norwegian Fit Futures cohort
Source: JBMR Plus. 2026 Aug 25;10(10):ziag143. doi: 10.1093/jbmrpl/ziag143 (PMC13549439; doi:10.1093/jbmrpl/ziag143)
Supplement: Supplementary file 1 [file supplementary_material__ziag143.zip › Supplementary Table S1 Mean total body lean mass and fat mass according to BMI trajectory group at each examination (FF1, FF2, and FF3). The Fit Future Study.docx]

**Supplementary Table S1** Mean total body lean mass and fat mass according to BMI trajectory group at each examination (FF1, FF2, and FF3). The Fit Future Study

|  | BMI trajectory |  | *FF1* *Mean (kg)* | *FF2 Mean (kg)* | *FF3* *Mean (kg)* |
| --- | --- | --- | --- | --- | --- |
| Females | Low-stable | Lean mass | 37.7 | 38.4 | 41.7 |
|  |  | Fat mass | 16.4 | 17.9 | 20.1 |
|  | Moderate-increasing | Lean mass | 39.3 | 39.9 | 45.0 |
|  |  | Fat mass | 26.5 | 28.6 | 34.8 |
|  | High-Stable | Lean mass | 43.7 | 45.2 | 53.4 |
|  |  | Fat mass | 41.2 | 45.8 | 48.8 |
| Males | Low-stable | Lean mass | 52.6 | 55.4 | 55.8 |
|  |  | Fat mass | 9.4 | 11.3 | 18.2 |
|  | Moderate-increasing | Lean mass | 55.0 | 57.6 | 61.1 |
|  |  | Fat mass | 21.9 | 25.0 | 29.5 |
|  | High-Stable | Lean mass | 63.9 | 64.6 | 72.7 |
|  |  | Fat mass | 44.5 | 47.9 | 54.7 |

*Values are presented as observed means (kg). Lean and fat mass are presented descriptively and were not included as covariates in the primary analyses.*
