# Supplementary material for: Associations between BMI trajectories and young adult bone: a 10-yr follow-up from the Norwegian Fit Futures cohort
Source: JBMR Plus. 2026 Aug 25;10(10):ziag143. doi: 10.1093/jbmrpl/ziag143 (PMC13549439; doi:10.1093/jbmrpl/ziag143)
Supplement: Supplementary file 1 [file supplementary_material__ziag143.zip › Supplementary Table S2.docx]

**Supplementary Table S2** Adjusted mean areal bone mineral density [aBMD] and bone mineral content [BMC] at FF1–FF3 by BMI trajectory group in females, The Fit Futures study

| Skeletal site | BMI trajectory | *FF1 Mean 95% CI* | *FF2 Mean 95% CI* | *FF3 Mean 95% CI* |  |
| --- | --- | --- | --- | --- | --- |
|  |  |  |  |  |  |
| Total body  aBMD (g/cm2) | Low-stable | 1.1123 [1.115, 1.130] | 1.139 [1.132, 1.144]] | 1.185 [1.177, 1.193] |  |
|  | Moderate-increasing | 1.185 [1.172, 1.198] | 1.197 [1.184, 1.211] | 1.251 [1.236, 1.266] |  |
|  | High-Stable | 1.223 [1.199, 1.249] | 1.223 [1.199, 1.249] | 1.293 [1.265, 1.322] |  |
| Total hip  aBMD (g/cm2) | Low-stable | 1.034 [1.022, 1.047] | 1.042 [1.030,1.055] | 1.022 [1.008, 1.035] |  |
|  | Moderate-increasing | 1.113 [1.091, 1.135] | 1.118 [1.096, 1.140] | 1.101 [1.077, 1.124] |  |
|  | High-Stable | 1.159 [1.119, 1.199] | 1.186 [1.145, 1.227] | 1.154 [1.109, 1.198] |  |
| Femoral neck  aBMD (g/cm2) | Low-stable | 1.042 [1.030, 1.055] | 1.048 [1.035, 1.060] | 1.017 [1.003, 1.030] |  |
|  | Moderate-increasing | 1.118 [1.086, 1.143] | 1.121 [1.100, 1.144] | 1.083 [1.060, 1.144] |  |
|  | High-Stable | 1.169 [1.119, 1.199] | 1.169 [1.128, 1.210] | 1.124 [1.078, 1.169] |  |
| Total body  BMC (g) | Low-stable | 2416.0 [2380.4, 2451.5] | 2496.5 [2461.2, 2531,9] | 2361.0 [2322.9, 2399.2] |  |
|  | Moderate-increasing | 2793.0 [2731.1, 2854.8] | 2873.3 [2810.4, 2936.2] | 2552.8 [2485.6, 2619.9] |  |
|  | High-Stable | 3018.4 [2903.0, 3133.7] | 3091.7 [2974.2, 3209.1] | 2287.7 [2558.3, 2817.2] |  |
| Total hip  BMC (g) | Low-stable | 31.0 [30.5, 31.5] | 31.3 [30.8, 31.8] | 31.2 [30.8, 31.7] |  |
|  | Moderate-increasing | 34.3 [33.5, 35.2] | 34.5 [33.7, 35.4] | 34.6 [33.7, 35.5] |  |
|  | High-Stable | 36.4 [34.9, 38.0] | 37.4 [35.9, 39.0] | 37.0 [35.3, 38.7] |  |
| Femoral neck  BMC (g) | Low-stable | 4.8 [4.7, 4.8] | 4.8 [4.7, 4.9] | 4.7 [4.6, 4.8] |  |
|  | Moderate-increasing | 5.3 [5.2, 5.5] | 5.3 [5.2, 5.4] | 5.2 [5.1, 5.3] |  |
|  | High-Stable | 5.5 [5.3, 5.8] | 5.6 [5.3, 5.8] | 5.4 [5.2, 5.7] |  |

*Values are adjusted mean estimates with 95% confidence intervals [CI] derived from linear mixed-effects models (Model 2). Models include time, BMI trajectory group, and their interaction. Estimates were adjusted for baseline age (FF1), pubertal timing, physical activity, smoking status, alcohol use, vitamin D status, and hormonal contraceptive use. A significant BMI trajectory × timepoint interaction was observed for total body BMC (p < 0.001), whereas no significant interactions were found for other skeletal sites (all p > 0.05).*
