# Supplementary material for: Associations between BMI trajectories and young adult bone: a 10-yr follow-up from the Norwegian Fit Futures cohort
Source: JBMR Plus. 2026 Aug 25;10(10):ziag143. doi: 10.1093/jbmrpl/ziag143 (PMC13549439; doi:10.1093/jbmrpl/ziag143)
Supplement: Supplementary file 1 [file supplementary_material__ziag143.zip › Supplementary Table S3.docx]

**Supplementary Table S3** Adjusted mean bone mineral density (aBMD) and bone mineral content (BMC) at FF1–FF3 by BMI trajectory group in males, The Fit Futures study

| Skeletal site | BMI trajectory | *FF1* *Mean [95% CI]* | *FF2 Mean [95% CI]* | *FF3* *Mean [95% CI]* |  |
| --- | --- | --- | --- | --- | --- |
|  |  |  |  |  |  |
| Total body  aBMD (g/cm^2^) | Low-stable | 1.164 [1.153, 1.174] | 1.203 [1.192, 1.215] | 1.284 [1.271, 1.296] |  |
|  | Moderate-increasing | 1.227 [1.211, 1.244] | 1.269 [1.251, 1.287] | 1.373 [1.353, 1.393] |  |
|  | High-Stable | 1.248 [1.205, 1.291] | 1.291 [1.242, 1.339] | 1.429 [1.380, 1.479] |  |
| Total hip  aBMD (g/cm^2^) | Low-stable | 1.096 [1.079, 1.112] | 1.114 [1.096, 1.132] | 1.071 [1.053, 1.090] |  |
|  | Moderate-increasing | 1.166 [1.140, 1.192] | 1.197 [1.169, 1.224] | 1.141 [1.111, 1.170] |  |
|  | High-Stable | 1.166 [1.098, 1.233] | 1.185 [1.111, 1.259] | 1.189 [1.113, 1.264] |  |
| Femoral neck  aBMD (g/cm^2^) | Low-stable | 1.086 [1.069, 1.102] | 1.114 [1.097, 1.132] | 1.049 [1.030, 1.068] |  |
|  | Moderate-increasing | 1.164 [1.138, 1.190] | 1.191 [1.163, 1.218] | 1.125 [1.095, 1.155] |  |
|  | High-Stable | 1.144 [1.078, 1.211] | 1.183 [1.109, 1.258] | 1.151 [1.075, 1.227] |  |
| Total body  BMC (g) | Low-stable | 2847.4 [2799.9, 2895.0] | 3054.4 [3003.1, 3105.6] | 3016.0 [2961.5, 3070.5] |  |
|  | Moderate-increasing | 3258.6 [3182.2, 3335.0] | 3503.8 [3423.7, 3583.9] | 3236.2 [3150.5, 3321.8] |  |
|  | High-Stable | 3350.8 [3154.4, 2547.3] | 3612.6 [3398.4, 3826.7] | 3544.5 [3326.3, 3762.8] |  |
| Total hip  BMC (g) | Low-stable | 38.8 [38.1, 39.6] | 39.9 [39.1, 40.6] | 39.2 [38.4, 40.0] |  |
|  | Moderate-increasing | 42.6 [41.4, 43.7] | 44.0 [42.8, 45.2] | 43.0 [41.7, 44.3] |  |
|  | High-Stable | 43.1 [40.2, 46.1] | 44.2 [40.9, 47.4] | 44.8 [41.5, 48.1] |  |
| Femoral neck  BMC (g) | Low-stable | 5.8 [5.7, 5.9] | 6.0 [5.9, 6.1] | 5.7 [5.6, 5.8] |  |
|  | Moderate-increasing | 6.4 [6.3, 6.6] | 6.6 [6.4, 6.8] | 6.3 [6.1, 6.5] |  |
|  | High-Stable | 6.3 [5.8, 6.7] | 6.5 [6.0, 7.0] | 6.3 [5.8, 6.8] |  |

*Values are adjusted mean estimates with 95% confidence intervals [CI] derived from linear mixed-effects models (Model 2). Models include time, BMI trajectory group, and their interaction. Estimates were adjusted for baseline age (FF1), pubertal timing, physical activity, smoking status, alcohol use, and vitamin D status. Significant BMI trajectory × time interactions were observed for total body aBMD and total body BMC (both p < 0.05), whereas no significant interactions were observed for other skeletal sites.*
